# A universally applicable toolbox for single-molecule quantification of chimeric antigen receptors using linker-resolved *d*STORM microscopy

**DOI:** 10.3389/fimmu.2026.1897225

**Published:** 2026-07-15

**Authors:** Josefine Michael, Peter Spieler, Fabio Toppeta, Leon Gehrke, Nicole Seifert, Björn Grams, Rick Seifert, Hermann Einsele, Michael Hudecek, Markus Sauer, Thomas Nerreter

**Affiliations:** 1 Chair of Cellular Immunotherapy, Department of Internal Medicine II, University Hospital Würzburg (UKW), Würzburg, Germany; 2 Department of Biotechnology and Biophysics, Biocenter, Am Hubland, University of Würzburg, Würzburg, Germany; 3 Department of Internal Medicine II, University Hospital Würzburg (UKW), Würzburg, Germany

**Keywords:** anti-CD19, CAR-T cell therapy, chimeric antigen receptor, *d*STORM, microscopy

## Abstract

Chimeric antigen receptor (CAR)-T cell therapies targeting CD19 have demonstrated remarkable clinical efficacy in B-cell malignancies. However, substantial differences exist in therapeutic outcomes, partly due to differences in CAR design and surface expression. Current methods for CAR detection, including flow cytometry, do not allow direct quantification of receptor density. Here, we establish a linker-targeted *direct* stochastic optical reconstruction microscopy (*d*STORM) approach for quantitative assessment of CAR surface expression across structurally diverse CD19 CAR-T cell products. By targeting conserved scFv linker regions, including (G4S)3 and Whitlow linkers, we enable antigen-independent detection using commercially available antibodies. We generated primary human T cells expressing constructs resembling approved CD19 CAR-T cell products and compared CAR detection by flow cytometry and *d*STORM. While flow cytometry enables detection, *d*STORM demonstrated superior sensitivity, allowing reliable visualization of CAR expression levels insufficient for flow cytometry. Direct CAR detection via linker-targeting antibodies revealed construct-dependent differences in CAR surface density, with a (G4S)3-containing construct exhibiting higher receptor densities compared to two Whitlow-based designs. *d*STORM resolved these differences more reliably at low expression levels, where flow cytometry yielded more CAR-negative events, suggesting that transfection marker staining and flow-based analyses may not fully capture CAR surface expression. Validation in clinically relevant CAR products underscores the robustness and versatility of this toolbox for CAR-specific staining across constructs. Overall, linker-targeted *d*STORM represents a highly sensitive and broadly applicable platform for quantification of CAR surface expression, offering new opportunities to understand how CAR design influences receptor density, spatial organization, and therapeutic function.

## 1 Introduction

Chimeric antigen receptor (CAR)-T cell therapy has transformed the treatment of B-cell malignancies and is now established as a standard of care. Seven CAR-T products have been approved by the U.S. Food and Drug Administration (FDA) and European Medicines Agency (EMA), including five products directed against Cluster of Differentiation 19 (CD19) for the treatment of leukemia and lymphoma (1, 2). Although these products recognize the same target antigen, they exhibit substantial differences in clinical efficacy, persistence, and toxicity. Beyond patient- and disease-specific factors, the CAR-T products themselves vary in the molecular design of their CAR receptors, which contributes to divergent clinical outcomes (3).

Quantitative assessment of CAR molecules at the cell surface remains insufficiently standardized. Current detection strategies primarily rely on genomic or fluorescence-based methods. Quantitative PCR (qPCR) and droplet digital PCR (ddPCR) determine vector copy number or transcript abundance but do not provide information on CAR protein expression or membrane localization, due to post-transcriptional regulation and protein turnover (4, 5). Flow cytometry enables protein-level detection and in addition to direct CAR staining, co expressed transfection markers such as truncated epidermal growth factor receptor (EGFRt) are frequently employed to identify modified cells. However, expression levels of the transfection marker and the CAR may differ due to independent regulation or trafficking (6). Common direct CAR staining approaches include staining with soluble antigen probes, anti Fab or anti-idiotype antibodies directed against the single-chain variable fragment (scFv), or Protein L binding to immunoglobulin light chains. Nonetheless, these strategies are reagent- and affinity-dependent, influenced by binding kinetics, epitope accessibility, and construct-specific architecture (6–8). Consequently, fluorescence intensity reflects binding behavior rather than receptor surface expression levels. To reduce antigen dependency, antibodies targeting conserved structural elements such as IgG hinge domains or flexible linker sequences such as Whitlow or glycine-serine repeats (G4S)3 linkers are used, enabling broader applicability across CAR constructs (9–11). However, these strategies rely on fluorescence signal intensity rather than quantifying receptor number at the cell surface.

The limited quantification of CAR surface density is particularly relevant as receptor surface expression levels and spatial organization shape immune signaling. In conventional T cells, the number and organization of T cell receptors (TCRs) determine activation thresholds, immune synapse formation, and downstream signaling strength (12, 13). Emerging evidence suggests that similar principles apply to CARs, where receptor density has been linked to tonic signaling, activation sensitivity, and the development of exhaustion. Excessive CAR expression can promote ligand independent signaling, whereas insufficient receptor density may impair antigen responsiveness. Thus, defining the number and density of CAR molecules at the plasma membrane is essential to understand how structural CAR design translates into functional outcome (14, 15).

Super-resolution microscopy enables visualization of membrane organization at nanometer resolution and encompasses single-molecule localization microscopy (SMLM) allowing reconstruction of molecular distributions beyond the diffraction limit. SMLM techniques include photoactivated localization microscopy (PALM), which relies on the sequential activation of fluorescent probes, DNA-based Point Accumulation for Imaging in Nanoscale Topography (DNA-PAINT), utilizing the transient binding of dye-labeled DNA strands to complementary target sequences, whereas direct stochastic optical reconstruction microscopy (*d*STORM) achieves stochastic localization of individual photoswitchable fluorophores (16). These approaches have fundamentally advanced the understanding of immune receptor organization, including T cell receptor clustering and synapse architecture but also of antigen recognition mechanisms across diverse immune cell types (12, 17).

Extending this concept to CAR-T cells, we have previously combined IgG4 hinge-targeting F(ab’)_2_ fragments with *d*STORM to enable quantification of CAR surface density for the first time. By targeting the extracellular hinge domain, this strategy allowed measurement at molecular level without reliance on antigen binding or scFv-specific reagents, representing an important conceptual advance in CAR imaging (18). However, hinge domains vary between clinically approved CAR constructs, including CD28- and CD8α-based hinges. IgG4 hinge-based detection strategies, while effective in certain constructs, are not applicable to CD28- or CD8α-based CARs. In contrast, a flexible linker connecting the VH and VL domains is an inherent structural feature of all scFv-based CARs. Since all commercially available CAR products and most research CARs contain either (G4S)3 or Whitlow linker sequences, targeting these conserved structural elements expands applicability beyond hinge-restricted strategies and enables broader cross-construct comparison. Here, we apply linker-targeted *d*STORM to directly quantify CAR surface density and nanoscale organization across structurally diverse constructs resembling FDA-approved CD19 CAR-T products using commercially available antibodies directed against (G4S)3 or Whitlow linker sequences.

## 2 Methods

### 2.1 Cell lines and cell culture media

K-562, Raji cell lines (ATCC) and TM-LCL [provided by courtesy of Prof. S. Riddell (19)] were cultured at 37 °C and 5% CO_2_ in RPMI-1640 medium (Thermo Fisher Scientific; 72400-054) supplemented with 10% (v/v) heat-inactivated fetal calf serum (FCS) and 100 U/mL penicillin/streptomycin (Thermo Fisher Scientific). K-562 CD19 OE were generated by lentiviral transduction with full-length *CD19*. Raji KO cells were generated by CRISPR/Cas9-mediated CD19 knockout. Cell lines stably expressing the EGFP-ffluc fusion protein were used in all experiments. Primary T cells were cultivated at 37 °C and 5% CO_2_ in RPMI-1640 medium supplemented with 10% (v/v) heat-inactivated human serum (HS), 100 U/mL penicillin/streptomycin and 0.05 mM β-mercaptoethanol.

### 2.2 Vector construction

*Sleeping beauty* (SB100X) vectors containing CAR-T cell constructs of three FDA approved CD19 CAR-T products (Tisagenlecleucel, Lisocabtagen-Maraleucel, Axicabtagen-Ciloleucel/Brexucabtagen-Autoleucel) were designed as previously described (20). All vectors contained EGFRt downstream of the CAR transgene separated with a T2A ribosomal skip element sequence for specific enrichment and depletion of the generated CAR-T cells (21).

### 2.3 Generation of human CAR-T cells

For the generation of human CAR-T cells, blood samples were collected from healthy donors obtained from leukocyte reduction chambers provided by the Department for Transfusion Medicine of the University Hospital Würzburg after written informed consent. CAR-T cells were generated as previously described, with detailed protocols provided in the **Supplementary Material** (19, 20).

### 2.4 Flow cytometry and data analysis

Data was collected on a Cytek^®^ Northern Lights™ (Cytek^®^ Bioscience) and analyzed using FlowJo V10.8.1 (FlowJo LLC). A complete list of fluorochrome-conjugated antibodies used in this study is provided in **Supplementary Table 1**. For self-labeling, the Whitlow linker antibody was conjugated as described in Section 2.6. Cells were stained as previously described (22). Detailed experimental procedures are provided in the **Supplementary Material**.

### 2.5 Functional characterization

For functional analyses, the proliferation-, cytotoxicity and cytokine secretion capacity were assessed. Regarding cytotoxicity, CD8^+^ CAR-T cells were co-cultured with EGFP-ffLuc^+^ tumor cells at indicated effector-to-target (E:T) ratios for 4 h in triplicates. Firefly D-luciferin (Biosynth; L-8220) was added at 150 µg/mL and tumor cell viability was quantified by bioluminescence using a Tecan Spark plate reader (Tecan). Specific lysis was calculated relative to control untransfected (UTD) T cells.

For effector cytokine secretion, CD4^+^ and CD8^+^ CAR-T cells were co-incubated with tumor cells at an E:T ratio of 4:1 for 24 h in triplicates. Medium (T cell only) and PMA/ionomycin were used as negative and positive controls, respectively. IFNγ concentrations in the co-culture supernatants were quantified by ELISA following the manufacturer’s protocol (BioLegend).

Proliferation was evaluated using the CellTrace™ CFSE dye. CD4^+^ and CD8^+^ T cells were labeled with 0.1 μM CFSE and co-incubated with irradiated tumor cells at a final E:T ratio of 4:1. Proliferation was quantified after 72 h by flow cytometry using the MACSQuant^®^ Analyzer 10.

### 2.6 Linker antibody conjugation for CAR detection

Purified Whitlow antibody was conjugated to Alexa Fluor 647 for CAR detection. A detailed conjugation protocol is provided in the **Supplementary Material**.

### 2.7 *d*STORM imaging

8 well chambered cover glass chambers (Cellvis; C8-1.5H-N) were treated with 1 M KOH (Roth; 9522.1) for 1 h, washed with dH2O, coated with a 1:4 dilution of PLL (Sigma-Aldrich; P4707) or PDL (Gibco; A38904-01) in PBS for 1 h and stored at 4 °C. 1x10^6^ T cells were washed twice with FACS buffer. Supernatant was removed, cells were resuspended in 160 µL FACS buffer and 20 µL of human TruStain FcX and incubated for 30 min at 4 °C. Conjugated antibody was adjusted to desired concentration of 10 µg/mL and incubated for 30 min at 4 °C. Stained cells were then washed four times with FACS buffer and twice with PBS. Washed cells were resuspended and transferred into coated chamber wells on ice. Cell suspension was carefully removed after 10 to 15 min and fixed using a dilution of 3% Formaldehyde (Roth; 4235.1) and 0.25% Glutaraldehyde (Sigma-Aldrich; G6257) in PBS for 15 min. Fixation solution was removed and washed three times with PBS. For *d*STORM measurements, an ONI Nanoimager S was used. PBS was removed and exchanged with imaging buffer (100 mM cysteamine hydrochloride (Sigma-Aldrich; M6500) in PBS, pH 7.4). Images were taken in total inner reflection fluorescence (TIRF) mode. Pixel size was 117 nm. 15000 frames at 10 ms exposure time were taken with a laser power of approximately 3.5 kW/cm². For final concentration experiments, an average of 245 (range 200-279) cells per condition was analyzed, whereas titration experiments included 25 cells per condition on average.

### 2.8 *d*STORM data analysis

*d*STORM cluster analysis was performed as previously described (18). Briefly, image reconstruction was performed with rapidSTORM 3.3 (23). Drift correction was performed by the linear drift correction tool of rapidSTORM 3.3. Selection of cell region of interests (ROIs) was performed with Napari and cross validated with brightfield images (24). For cluster analysis, a custom localization analysis tool LOCAN (25) was used, which employs a density-based spatial clustering of applications with noise (DBSCAN) algorithm. Cluster parameters of DBSCAN were set to minPoints = 3 and epsilon = 20 nm according to Ebert et al. (26). Chosen cluster parameters allow quantification of localizations within a fixed distance, which provides information about receptor numbers on the cell surface and remove random localizations gathered by autofluorescence or camera read-out noise (26, 27).

### 2.9 Data and statistical analysis

Data analysis was performed in Excel (Microsoft Office, version 2408). GraphPad Prism software (GraphPad, version 10.0.1) was used for generating graphs and performing statistical analysis. Flow cytometry data was analyzed using FlowJo™ v10.10.0 Software (BD Biosciences). Individual tests are indicated in the respective figure legends. P-values are stated exactly or represented by: **** = P ≤ 0.0001; *** = P ≤ 0.001; ** = P ≤ 0.01; * = P ≤ 0.05; ns = P > 0.05. CAR localization clusters/µm² are described as mean ± SEM in the text. Figures were prepared using PowerPoint (Microsoft Office, version 2408) or BioRender.com. OpenAI’s ChatGPT (GPT-5-mini architecture; knowledge cutoff: 2025-08) was used solely to improve readability and check grammar. All outputs were reviewed and validated by the authors.

## 3 Results

### 3.1 Generation of primary T cells expressing distinct clinically derived CD19 CAR constructs

**Figure 1:**
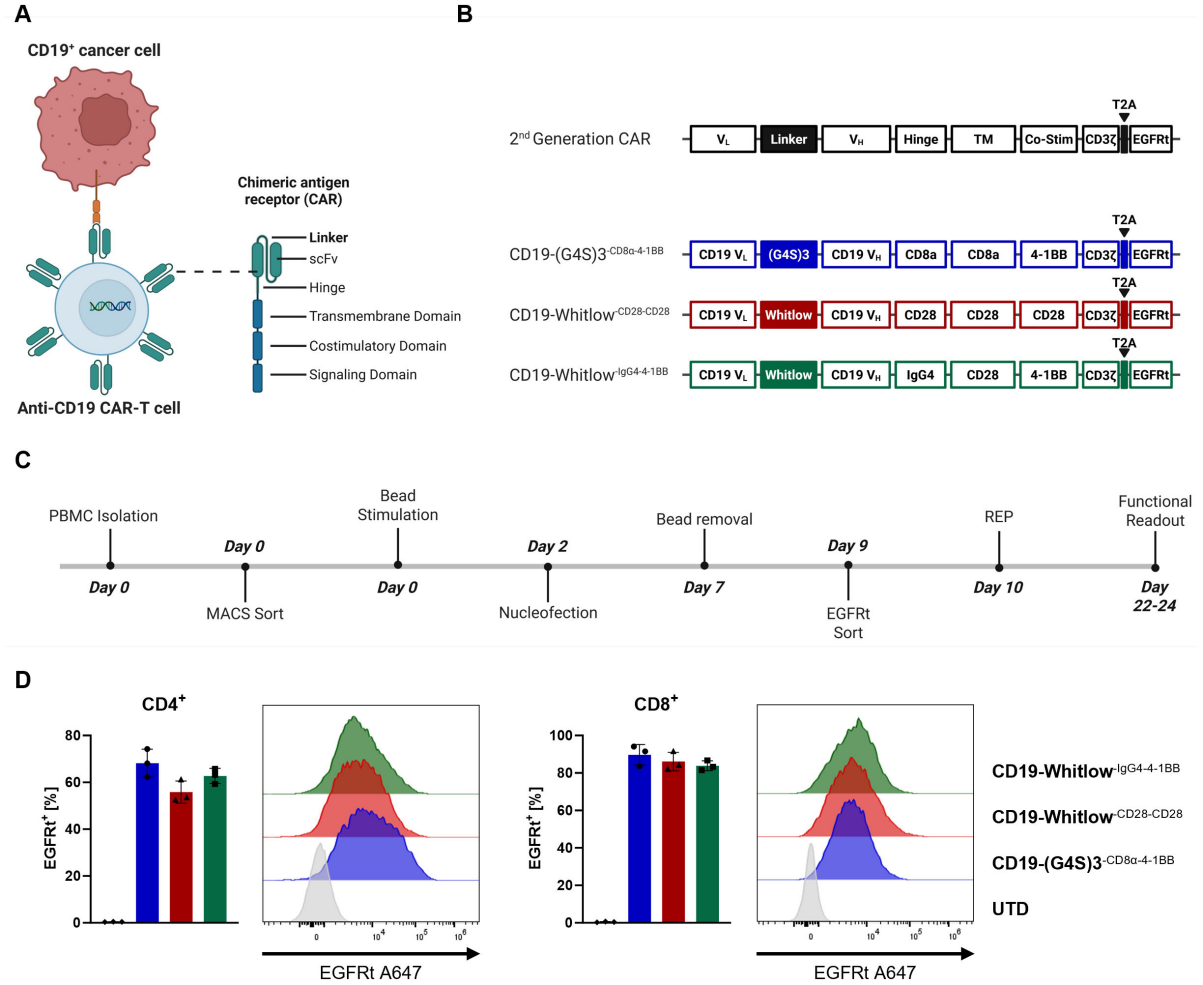
Generation of primary T cells expressing distinct clinically derived CD19 CAR constructs. **(A)** Schematic depiction of CD19 CAR-T cells engaging CD19-positive tumor cells via the extracellular scFv, which is connected by a flexible linker. **(B)** Schematic illustration of CAR constructs used for human CAR-T cell generation. The constructs encode an FMC63-derived scFv connected via a (G4S)3 or Whitlow linker, followed by hinge, transmembrane, costimulatory and signaling domains. The transgene cassette includes a truncated EGFR as transfection marker, which is separated by a T2A sequence. **(C)** Experimental workflow for CAR-T cell generation and expansion. **(D)** Expression of EGFRt in CAR-T cells compared to untransfected T cells. Data shown are mean ± SD for n = 3 independent donors. Representative histogram plots show CD4^+^ and CD8^+^ T/CAR-T cells from one donor.

Three structurally distinct CD19 CAR constructs corresponding to clinically approved CAR-T products were reconstructed and expressed in primary human T cells. Currently, five CD19 CAR-T therapies are approved by FDA and EMA, with four of them using the same targeting domain (FMC63 scFv). Two of these products share an identical CAR architecture and differ only in their clinical manufacturing process. The constructs used in this study represent clinically approved products comprising tisagenlecleucel (CD19-(G4S)3^-CD8α-4-1BB^), lisocabtagen-maraleucel (CD19-Whitlow^-IgG4-4-1BB^) and axicabtagen-ciloleucel/brexucabtagen-autoleucel (CD19-Whitlow^-CD28-CD28^), the latter two being structurally identical at the level of the CAR. All constructs contain either a (G4S)3 or Whitlow linker, while differing in hinge, transmembrane and costimulatory domains (**Figures 1A, B**).

The more recently developed CD19 CAR obecabtagene autoleucel incorporates an affinity-tuned antigen-binding domain and therefore differs at the level of the scFv sequence. However, this scFv-based CAR retains a flexible V_H_-V_L_ linker, typically composed of glycine-serine repeats. While the present study focuses on the classical FMC63-based CAR architectures, the linker-targeted approach described here is transferable to all CAR designs where the antigen-binding domain is connected via a (G4S)3 or Whitlow linker. To enable direct comparison of CAR density, all constructs were expressed under standardized experimental conditions (**Figure 1C**). Each construct co-expresses the surface transfection marker EGFRt via a T2A self-cleaving peptide, enabling enrichment of CAR-positive cells and ensuring comparable expression levels across constructs (**Figure 1D**).

### 3.2 Direct quantification of CAR surface expression by linker-specific staining using flow cytometry and *d*STORM

**Figure 2:**
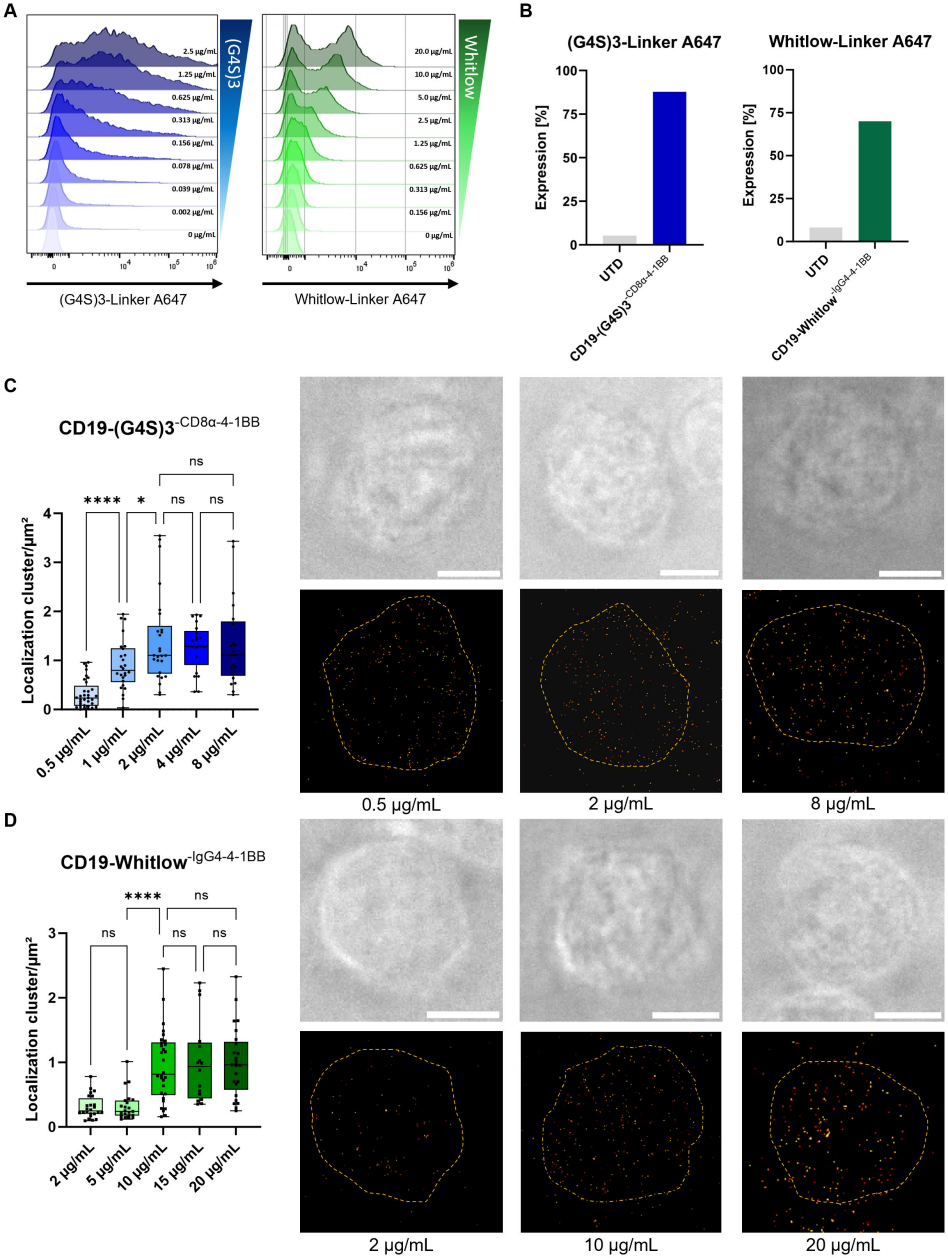
Direct quantification of CAR surface expression by linker-specific staining using flow cytometry and *d*STORM. **(A)** Antibody titration for linker-specific detection of CAR constructs by spectral flow cytometry. Representative histograms showing staining of EGFRt-sorted CAR-T cells using commercially available Alexa Fluor 647-conjugated antibodies targeting the (G4S)3- (left, blue; Cell Signaling Technology, E7O2V) or Whitlow (right, green; Miltenyi Biotec, REA1400) linker. Representative histograms are shown from one donor. **(B)** Quantification of linker-positive CAR-T cells by spectral flow cytometry. Bar graph showing the percentage of linker-positive cells in CAR-T cell products or untransfected T cells (UTD) following staining with (G4S)3- or Whitlow-specific antibodies at the selected final working concentrations (2.5 μg/mL and 15 μg/mL respectively). Representative graphs are shown from one donor. **(C, D)** Antibody titration for linker-specific CAR detection by *d*STORM super-resolution microscopy. Untransfected (UTD) and CAR-T cells were stained with increasing concentrations of (G4S)3- (**(C)**, blue; Cell Signaling Technology, E7O2V) or Whitlow-specific (**(D)**, green; Miltenyi Biotec, REA1400) antibodies. Each point represents an individual cell from one representative donor. For each antibody concentration, 21–33 cells were analyzed in **(C)** and 16–30 cells were analyzed in **(D)**. Box plots indicate the median and interquartile range, with whiskers showing the minimum to maximum values. Statistical analysis was performed using Brown-Forsythe and Welch ANOVA with Welch correction. Right panels: representative *d*STORM images of CAR-T positive cells stained with various concentrations. Scale bars = 5 µm. Significance indicated as: ****P ≤ 0.0001; *P ≤ 0.05; ns = P > 0.05.

We next sought to determine optimal staining conditions for antibodies targeting either the (G4S)3- or Whitlow linker. Antibody titrations were therefore performed on EGFRt-sorted CAR-T cells for both spectral flow cytometry and *d*STORM (**Figure 2**). Titrations were initiated at twice the manufacturer’s recommended concentration, followed by serial dilutions. Final working concentrations were selected based on saturation of the respective linker epitopes while minimizing background staining. The initially tested commercially available Whitlow linker antibody (Cell Signaling; Clone E3U7Q) showed good separation of positive and negative populations at higher concentrations in flow cytometry. However, due to the low stock concentration of 6.25 µg/mL provided by the manufacturer, this antibody was not suitable for repeated flow cytometric staining and was therefore excluded from further experiments (**Supplementary Figure 1A**). Instead, an alternative Whitlow specific antibody (Miltenyi Biotec; Clone REA1400) was used and conjugated in-house to Alexa Fluor 647. While this antibody enabled detection of the Whitlow linker at higher concentrations, it exhibited increased background staining across the tested dilution range (**Figures 2A, B**; **Supplementary Figure 1B**). Based on the titration results, final working concentrations for flow cytometry were set to 2.5 µg/mL for (G4S)3-specific antibody and 15 µg/mL for the Whitlow-specific antibody.

For optimal staining conditions of *d*STORM experiments, we first validated minimal unspecific background staining with (G4S)3 and Whitlow linker antibodies on untransfected CD8^+^ T cells (**Supplementary Figure 1C**). Next, we performed *d*STORM of EGFRt-sorted CD8^+^ CAR-T cells at various antibody concentration conditions (**Figure 2C**). *d*STORM shows plateauing of CAR surface expression for CD19-(G4S)3^-CD8a-4-1BB^ and corresponding antibody between 2.0 µg/mL and 8.0 µg/mL and for CD19-Whitlow^-IgG4-4-1BB^ between 10 µg/mL and 20 µg/mL with in-house conjugated antibody, while the commercially available antibody did not reach saturation at practical working dilutions above 1:5 (1 µg/mL) (**Supplementary Figure 1D**). We therefore determined a (G4S)3 antibody concentration of 2.5 µg/mL and a Whitlow-linker antibody concentration of 15 ug/mL to be ideal for subsequent *d*STORM experiments, which was in accordance with our flow cytometry titration data. To illustrate the importance of optimized labeling conditions to correctly depict differences in CAR surface expression, we highlight reconstructed *d*STORM images at saturated or suboptimal antibody concentrations (**Figure 2D**).

### 3.3 Subset-dependent CAR surface expression and construct-associated differences in nanoscale clustering revealed by linker-specific staining

**Figure 3:**
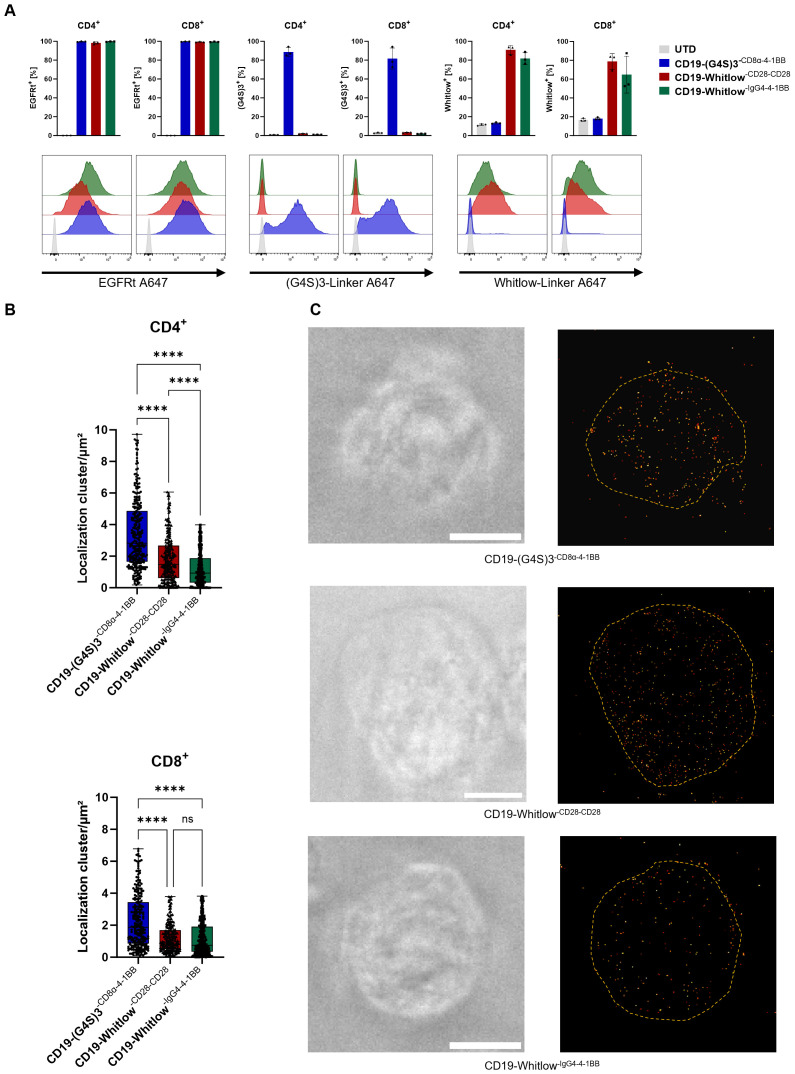
Subset-dependent CAR surface expression and construct-associated differences in nanoscale clustering revealed by linker-specific staining. **(A)** Flow cytometric analysis of CAR expression in CD4^+^ and CD8^+^ T cell subsets following two rounds of enrichment to ensure equal EGFRt expression levels. Top: CAR expression was assessed either indirectly via staining of the EGFRt transfection marker or directly via linker-specific detection using antibodies targeting the (G4S)3 or Whitlow linker. Data are presented as mean ± SD from n = 3 independent donors. Bottom: Representative histograms showing EGFRt and linker-specific staining ((G4S)3 and Whitlow) in untransfected (UTD) and CAR-T cell populations from one of the three donors. **(B)** *d*STORM-based quantification of CAR nanoscale organization following two rounds of enrichment to ensure equal EGFRt expression levels. CAR cluster density (clusters/µm²) was determined using linker-specific antibodies. Each point represents an individual cell from one of three independent donors. Box plots indicate the median and interquartile range, with whiskers showing the minimum to maximum values. For the upper graph, 216–279 cells, for the lower graph, 200–269 cells per condition were analyzed. Boxed-line indicates the median. Statistical analysis was performed using Brown-Forsythe and Welch ANOVA with Welch correction. **(C)** Representative bright field and *d*STORM images of CAR-T positive cells stained with the final working concentration of 2.5 μg/mL ((G4S)3) and 15 μg/mL (Whitlow). Scale bars = 5 µm. Significance indicated as: ****P ≤ 0.0001; ns = P > 0.05.

To further characterize surface expression of CARs resembling clinically approved CD19 CAR-T cell products, we compared spectral flow cytometry with *d*STORM. Although transfection marker expression was generally high across the analyzed samples, direct staining of the CAR linker sequences suggested a lower proportion of CAR-positive T cells when using flow cytometry for assessment (**Figure 3A**). Flow cytometry analysis further revealed minor differences between CD4^+^ and CD8^+^ T cell subsets. In particular, EGFRt transfection marker expression was modestly reduced, as indicated by decreased MFI in CD4^+^ T cells compared to CD8^+^ T cells, independent of the CAR linker type (**Supplementary Figure 2A**). In contrast, direct detection of the CAR linker sequences demonstrated lower CAR surface expression in CD8^+^ compared to CD4^+^ T cells, as reflected by both the percentage of CAR-positive cells and the MFI (**Figure 3A**, **Supplementary Figure 2A**). Furthermore, direct staining and visualization of the CAR linker regions indicated higher CAR expression in the construct containing a (G4S)3 linker compared to those containing the Whitlow linker. Notably, these differences were not apparent when assessing the transfection marker expression.

Further analysis via *d*STORM revealed differences in CAR surface expression between both, subsets and constructs: In CD4^+^ cells, CD19-(G4S)3^-CD8α-4-1BB^ exhibited the highest number of CARs present on the surface with approximately (3.41 ± 0.13 localization cluster/µm²) followed by CD19-Whitlow^-CD28-CD28^ with (1.84 ± 0.10 localization cluster/µm²) and CD19-Whitlow^-IgG4-4-1BB^ with (1.22 ± 0.07 localization cluster/µm²) (**Figures 3B, C**; **Supplementary Figure 2B**). In CD8^+^ cells, CD19-(G4S)3^-CD8α-4-1BB^ showed the highest level of CAR expression as well (2.28 ± 0.11 localization cluster/µm²), followed by CD19-Whitlow^-CD28-CD28^ with (1.22 ± 0.06 localization cluster/µm²) and CD19-Whitlow^-IgG4-4-1BB^ with (1.18 ± 0.06 localization cluster/µm²). Overall, CD4^+^ CAR-T cells exhibited higher CAR expression than CD8^+^ cells, and *d*STORM demonstrated greater sensitivity than flow cytometry for detecting CAR density, as indicated by a lower proportion of CAR-negative cells (**Supplementary Figures 2C, D**).

### 3.4 Distinct CAR designs and receptor densities shape functional responses and phenotypic states in CD19 CAR-T cells

**Figure 4:**
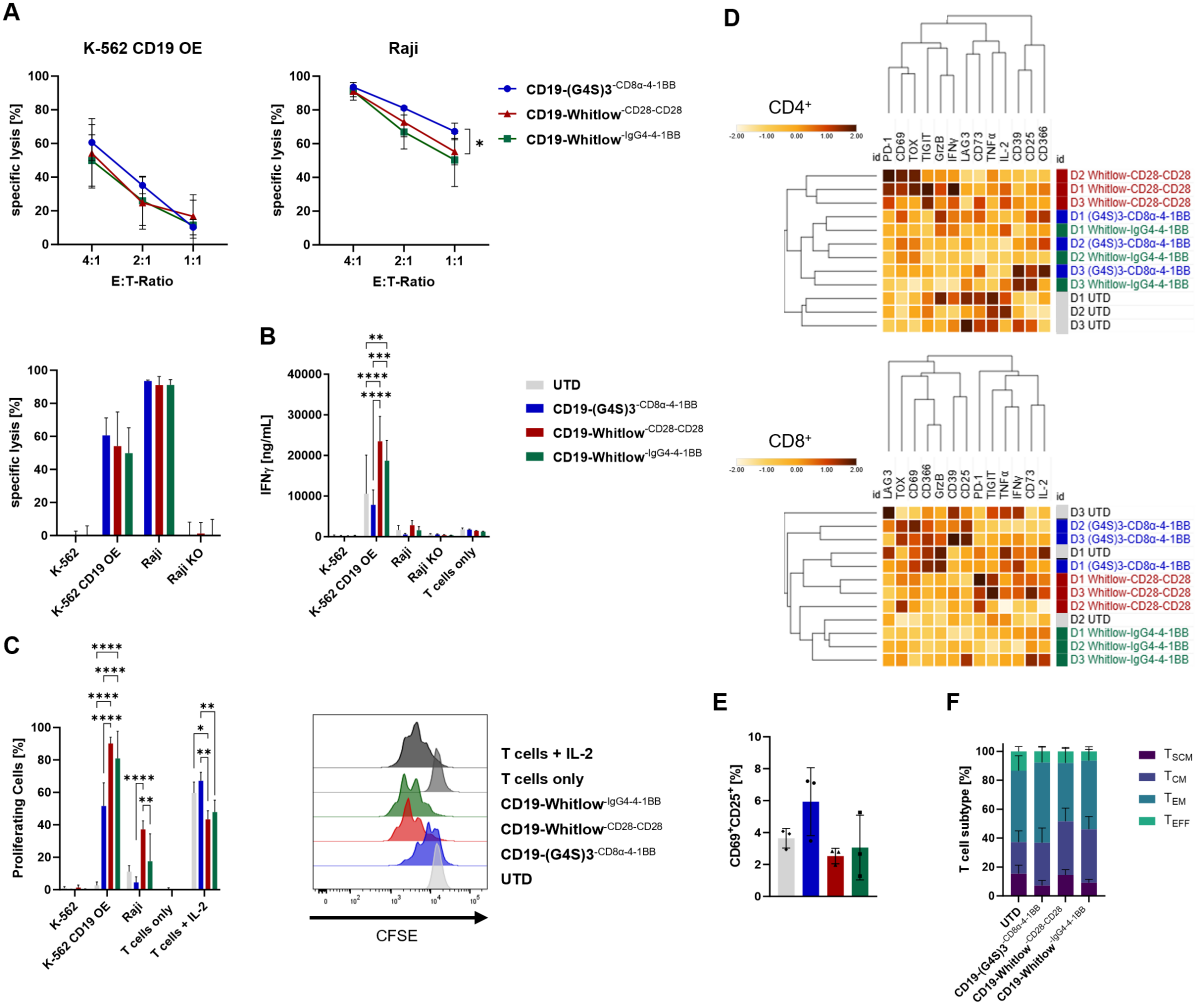
Distinct CAR designs and receptor densities shape functional responses and phenotypic states in CD19 CAR-T cells. **(A-C)** Functional characterization of structurally distinct CD19 CAR-T cell constructs in response to CD19^+^ target cells. Statistical analysis was performed using one-way ANOVA followed by Tukey’s multiple comparisons test. **(A)** Specific lysis of CD19^+^ tumor cell lines at indicated E:T ratios after 4 h co-culture with CD8^+^ CD19 CAR-T cells. **(B)** IFNγ secretion after 24 h co-culture of CD8^+^ CD19 CAR-T cells or untransfected (UTD) T cells with CD19^+^ tumor cell lines. **(C)** Proliferation of CD8^+^ CD19 CAR-T cells compared with untransfected (UTD) T cells after 72 h co-culture with indicated tumor cells (E:T 4:1) shown as percentage of proliferating cells and representative histograms. **(D)** Unsupervised hierarchical clustering and heatmap of flow cytometry phenotypes. Clustering was performed using Euclidean distance and average linkage in Morpheus, with CD4^+^ T/CAR-T cells displayed in the top panel and CD8^+^ T/CAR-T cells in the bottom panel. **(E)** Activation shown as frequency of CD25^+^ CD69^+^ double-positive CD8^+^ CD19 CAR-T cells or untransfected (UTD) T cells. **(F)** Relative frequencies of CAR-T cell phenotypes for CD8^+^ CD19 CAR-T cells or untransfected (UTD) T cells. Phenotypes are classified as T_SCM_ (stem cell memory-like), T_CM_ (central memory-like), T_EM_ (effector memory-like) and T_EFF_ (effector-like). Data are presented as mean ± SD for n = 3 independent donors. Significance indicated as: ****P ≤ 0.0001; **P ≤ 0.01; *P ≤ 0.05.

To compare functional properties across the structurally distinct CD19 CAR constructs and further investigate the functional consequences of the distinct receptor densities identified by *d*STORM, we performed comprehensive phenotypic and functional analyses of the different CAR-T cell products. All CAR-T cell constructs exhibited robust and antigen-specific cytotoxicity, effector molecule secretion, and proliferative capacity in response to CD19^+^ target cell lines, and target cell-dependent differences in response dynamics were observed. K562-CD19 OE cells were eliminated more gradually, which was accompanied by higher IFNγ secretion and increased proliferation, whereas Raji cells were rapidly cleared, resulting in comparatively lower IFNγ production and reduced proliferative responses. Among the constructs, CD19-Whitlow^-CD28-CD28^ CAR-T cells exhibited significantly increased proliferation in the presence of CD19^+^ tumor cells compared to the other CAR designs, indicating enhanced functional responsiveness under antigen stimulation, consistent with the known potent signaling capacity of CD28-based CAR constructs (**Figures 4A-C**; **Supplementary Figures 2E, F**).

Unsupervised clustering based on flow cytometric phenotyping revealed distinct patterns between CD4^+^ and CD8^+^ T cell subsets (**Figure 4D**). Within the CD4^+^ compartment, clustering was primarily associated with the costimulatory domain, with CD19-Whitlow^-CD28-CD28^ CAR-T cells displaying elevated expression of activation and exhaustion markers, including PD-1, CD69, TOX, and TIGIT, accompanied by increased Granzyme B and IFNγ levels. In contrast, within the CD8^+^ subset, CD19-(G4S)3^-CD8α-4-1BB^ CAR-T cells showed higher expression of activation- and exhaustion-associated markers such as TOX, CD69, CD366, and CD25. In the absence of exogenous stimulation, CAR-T cells within the CD4^+^ subset exhibited increased frequencies of cells co-expressing the early and late activation markers CD69 and CD25. A similar trend was observed in CD8^+^ T cells, but was restricted to constructs containing the (G4S)3 linker (**Figure 4E**; **Supplementary Figure 2G**). T cell memory subset distributions remained largely comparable across constructs in both, CD4^+^ and CD8^+^ populations (**Figure 2F**; **Supplementary Figure 2H**).

Our results show that linker-targeted *d*STORM provides a sensitive and quantitative method for measuring CAR density on human primary T cells. This approach is effective across structurally diverse CD19 CARs, as well as any CAR construct incorporating either a (G4S)3 or Whitlow linker.

## 4 Discussion

In this study, we establish linker-targeted *d*STORM as a sensitive and broadly applicable method for direct quantification of CAR surface density on primary human T cells. By targeting conserved structural elements within the scFv, like the (G4S)3 and Whitlow linker, this approach enables independent cross-construct comparison (10–12, 28). In contrast to conventional fluorescence-based methods, *d*STORM allows quantification of receptor surface expression levels at the basal plasma membrane, providing information on the CAR surface expression density or number of CAR molecules per cell rather than relative signal intensity, which enables a much more robust cross comparison of CAR surface expression between different CAR constructs, designs and laboratories. This broadly applicable toolbox for CAR detection uses commercially available antibodies against (G4S)3 or Whitlow linkers, enabling standardized analysis across diverse CAR designs. Importantly, we validate this approach across multiple clinically relevant CD19 CAR constructs. This is particularly relevant in the context of product characterization, where quantitative differences in CAR surface expression may contribute to functional heterogeneity and clinical performance.

Such considerations are in line with recent perspectives highlighting imaging approaches as powerful tools to study CAR-T cell behavior across multiple spatial scales (29). While clinical and preclinical imaging modalities such as positron emission tomography (PET), magnetic resonance imaging (MRI), and optical imaging techniques enable non-invasive tracking of CAR-T cell biodistribution, expansion, and tumor infiltration *in vivo*, they do not provide direct information on receptor organization or abundance at the molecular level. In this context, super-resolution imaging approaches such as *d*STORM complement these modalities by enabling nanoscale analysis of CAR surface expression and organization, thereby providing mechanistic insight into how construct design and cellular context influence receptor density and potentially downstream signaling (29).

Complementary to *in vivo* imaging approaches, flow cytometry is the most widely used *ex vivo* method for quantifying CAR expression at the single-cell level. While flow cytometry remains a robust and widely used method for CAR detection, our data show that *d*STORM especially exceeds at low target expression levels compared to flow cytometry and further enables quantitative insights at single-molecule level and extends the usable range of certain reagents. For example, one clone (Miltenyi Biotec; REA1400) yielded quantifiable signals in *d*STORM even at lower concentrations than flow cytometry, whereas another clone (Cell Signaling; E3U7Q) performed well with both techniques but usage by flow cytometry was limited by availability or concentration. Consistent with this, flow cytometry yielded more CAR-negative events, reflecting its lower sensitivity for detecting low CAR expression (**Figure 2**; **Supplementary Figures 1**, **2D**).

Recent studies underline the importance of advanced imaging methods for studying CAR expression at the proteomic level across different expression levels (5). CAR surface density, tumor antigen density, and CAR affinity play crucial roles in CAR downmodulation and, consequently, in the efficacy of CAR-T cell therapy (17, 18, 29). While several studies suggest that reduced CAR expression may improve therapeutic outcomes, the reported findings remain inconsistent (30–33). Moreover, information on ultra-low antigen expression levels on tumor cells may provide important insights for the clinical administration of CAR-T cell therapies (17). As precise control of CAR protein density on the T-cell membrane is critical for optimizing CAR-T cell products, establishing standardized methodological approaches for the reliable and cross-comparable detection of dynamic surface expression levels is essential for the field.

A key finding of our study is the ability to resolve a broad range of CAR expression levels across clinically relevant constructs. Using this approach, we demonstrate that CAR surface expression levels depend to a certain extent on CAR design. In particular, the (G4S)3-containing construct showed higher CAR surface densities compared to Whitlow-based constructs (**Figure 3B**), which coincides with the overall trend in flow cytometry data (**Figure 3A**, **Supplementary Figure 2A**). These differences were not reflected by EGFRt expression (**Figure 3A**), underscoring that commonly used transfection markers are insufficient for determining CAR surface abundance. Instead, our data emphasize the importance of direct protein-level quantification. However, cross-construct comparisons should be interpreted with caution, since potential differences in staining reagents, including antibody affinity, epitope accessibility, steric effects, labeling efficiency, as well as differences in antibody clones and staining concentrations could influence the results.

We observed subset-specific differences in CAR expression between CD4^+^ and CD8^+^ T cells by *d*STORM imaging in alignment with flow cytometry data (**Figure 3**; **Supplementary Figure 1**). Our findings here build on and extend our previous work, where we demonstrated CAR quantification using hinge-targeting strategies and showed that T cell subsets differentially regulate receptor expression and may thereby contribute to functional heterogeneity in CAR-T cell products (18). Both findings also provide a compact range of CAR surface expression across diverse CAR designs and diverse direct CAR labeling methods. Compared to alternative CAR detection strategies, such as antigen-based staining, anti-idiotype antibodies, or Protein L binding, linker-targeting offers several advantages, including independence from antigen binding, reduced susceptibility to affinity-related biases, and broad applicability across constructs (7, 8, 34, 35). While hinge-targeting approaches share some of these benefits, their applicability across diverse CAR designs can be restricted by structural variability of hinge regions, whereas linker-targeting provides a more conserved and thus more broadly applicable alternative.

Despite these strengths, several limitations should be considered. First, *d*STORM is inherently lower throughput, more technically and time demanding than flow cytometry, requiring specialized instrumentation and computational analysis, which may limit its broader application. Although on average, a comparatively large amount of 245 individual cells per condition were analyzed in final concentration experiments using *d*STORM, the lower throughput and overall reduced number of cells have to be taken into account when interpreting expression levels of overall cell populations. Second, linker accessibility may vary depending on CAR conformation or membrane context. Additionally, different antibody clones targeting the same linker sequence (e.g. REA1400 versus E3U7Q for Whitlow linkers) can differ in affinity, background staining, and suitability for specific applications, emphasizing the need for careful validation.

Although we show clear differences in CAR surface density between constructs, the functional consequences of these differences remain only partially understood, as they may also be influenced by differences in other structural elements of the CAR, including the hinge, transmembrane, and costimulatory domains. While the functional assays indicate robust activity across all constructs (**Figure 4**; **Supplementary Figure 2**), our data provide a basis for future studies using CAR designs that enable receptor density to be experimentally separated from receptor architecture, and to directly link nanoscale CAR organization and density to signaling strength, persistence, and exhaustion phenotypes (4, 36, 37).

In conclusion, linker-targeted *d*STORM enables direct and quantitative assessment of CAR surface expression across a wide dynamic range and diverse CAR designs. In combination with flow cytometry, this approach provides a complementary framework for robust CAR detection and characterization. By enabling direct quantification of CAR surface expression numbers and expanding the usable antibody repertoire, linker-targeted *d*STORM represents a valuable addition to the methodological toolbox for studying CAR-T cell products and their functional properties.

## Data Availability

The original contributions presented in the study are included in the article/**Supplementary Material**. Further inquiries can be directed to the corresponding author.

## References

[1] Braendstrup P Levine BL Ruella M . The long road to the first Fda approved gene therapy: Chimeric antigen receptor T cells targeting Cd19. Cytotherapy. (2020) 22:57–69. doi: 10.1016/j.jcyt.2019.12.004 32014447 PMC7036015

[2] Administration USFaD . Approved cellular and gene therapy products (2026). Available online at: https://www.fda.gov/vaccines-blood-biologics/cellular-gene-therapy-products/approved-cellular-and-gene-therapy-products (Accessed May 05, 2026).

[3] Bock TJ Colonne CK Fiorenza S Turtle CJ . Outcome correlates of approved Cd19-targeted Car T cells for large B cell lymphoma. Nat Rev Clin Oncol. (2025) 22:241–61. doi: 10.1038/s41571-025-00992-5 39966627

[4] Turicek DP Giordani VM Moraly J Taylor N Shah NN . Car T-cell detection scoping review: An essential biomarker in critical need of standardization. J Immunother Cancer. (2023) 11. doi: 10.1136/jitc-2022-006596 37217245 PMC10230989

[5] Hinckley-Boned A Barbero-Jiménez C Tristán-Manzano M Maldonado-Perez N Hudecek M Justicia-Lirio P . Tailoring Car surface density and dynamics to improve Car-T cell therapy. J Immunother Cancer. (2025) 13. doi: 10.1136/jitc-2024-010702 40300856 PMC12049969

[6] Chen P-H Raghunandan R Morrow JS Katz SG . Finding your Car: The road ahead for engineered T cells. Am J Pathol. (2024) 194:1409–23. doi: 10.1016/j.ajpath.2024.04.002 38697513 PMC11284763

[7] Cheng J Mao X Chen C Long X Chen L Zhou J . Monitoring anti‐Cd19 chimeric antigen receptor T cell population by flow cytometry and its consistency with digital droplet polymerase chain reaction. Cytometry A. (2023) 103:16–26. doi: 10.1002/cyto.a.24676 35875964 PMC10087589

[8] Schanda N Sauer T Kunz A Hückelhoven-Krauss A Neuber B Wang L . Sensitivity and specificity of Cd19.Car-T cell detection by flow cytometry and Pcr. Cells. (2021) 10:3208. doi: 10.3390/cells10113208 34831430 PMC8621201

[9] Sievers SA Kelley KA Astrow SH Bot A Wiltzius JJ . Abstract 1204: Design and development of anti-linker antibodies for the detection and characterization of Car T cells. Cancer Res. (2019) 79:1204. doi: 10.1158/1538-7445.AM2019-1204 30573519

[10] Grahnert A Seiffert S Wenk K Schmiedel D Boldt A Vucinic V . Evaluation of anti-Car linker Mabs for Car T monitoring after Bites/Bsabs and Car T-cell pretreatment. Biomedicines. (2024) 12:1641. doi: 10.3390/biomedicines12081641 39200107 PMC11351819

[11] Schindler K Ruppel KE Müller C Koehl U Fricke S Schmiedel D . Linker-specific monoclonal antibodies present a simple and reliable detection method for Scfv-based Car Nk cells. Mol Ther Methods Clin Dev. (2024) 32:101328. doi: 10.1016/j.omtm.2024.101328 39286335 PMC11403257

[12] Hu Y Huang J . The chimeric antigen receptor detection toolkit - Pubmed. Front Immunol. (2020) 11. doi: 10.3389/fimmu.2020.01770 32849635 PMC7431616

[13] Yokosuka T Saito T . The immunological synapse, Tcr microclusters, and T cell activation. In: Saito T Batista FD , editors. Immunological Synapse. Springer, Berlin, Heidelberg (2010). p. 81–107. 10.1007/978-3-642-03858-7_5 19960310

[14] Dong R Libby KA Blaeschke F Fuchs W Marson A Vale RD . Rewired signaling network in T cells expressing the chimeric antigen receptor (Car). EMBO J. (2020) 39:EMBJ2020104730. doi: 10.15252/embj.2020104730 32643825 PMC7429742

[15] Rodriguez-Marquez P Calleja-Cervantes ME Serrano G Oliver-Caldes A Palacios-Berraquero ML Martin-Mallo A . Car density influences antitumoral efficacy of Bcma Car T cells and correlates with clinical outcome. Sci Adv. (2022) 8:eabo0514. doi: 10.1126/sciadv.abo0514 36179026 PMC9524842

[16] Lelek M Gyparaki MT Beliu G Schueder F Griffié J Manley S . Single-molecule localization microscopy. Nat Rev Methods Primers. (2021) 1:39. doi: 10.1038/s43586-021-00038-x 35663461 PMC9160414

[17] Nerreter T Letschert S Götz R Doose S Danhof S Einsele H . Super-resolution microscopy reveals ultra-low Cd19 expression on myeloma cells that triggers elimination by Cd19 Car-T. Nat Commun. (2019) 10:3137. doi: 10.1038/s41467-019-10948-w 31316055 PMC6637169

[18] Gehrke L Seifert N Spieler P Verbruggen C Seifert R Toppeta F . Direct visualization of chimeric antigen receptors on primary human T cells using Dstorm super-resolution microscopy. Front Immunol. (2025) 16:1632823. doi: 10.3389/fimmu.2025.1632823 40821773 PMC12354347

[19] Riddell SR Greenberg PD . The use of anti-Cd3 and anti-Cd28 monoclonal antibodies to clone and expand human antigen-specific T cells. J Immunol Methods. (1990) 128:189–201. doi: 10.1016/0022-1759(90)90210-m 1691237

[20] Monjezi R Miskey C Gogishvili T Schleef M Schmeer M Einsele H . Enhanced Car T-cell engineering using non-viral Sleeping Beauty transposition from minicircle vectors. Leukemia. (2017) 31:186–94. doi: 10.1038/leu.2016.180 27491640

[21] Hudecek M Sommermeyer D Kosasih PL Silva-Benedict A Liu L Rader C . The non-signaling extracellular spacer domain of chimeric antigen receptors is decisive for *in vivo* antitumor activity. Cancer Immunol Res. (2015) 3:125–35. doi: 10.1158/2326-6066.CIR-14-0127 25212991 PMC4692801

[22] Feucht J Sun J Eyquem J Ho YJ Zhao Z Leibold J . Calibration of Car activation potential directs alternative T cell fates and therapeutic potency. Nat Med. (2019) 25:82–8. doi: 10.1038/s41591-018-0290-5 30559421 PMC6532069

[23] Wolter S Löschberger A Holm T Aufmkolk S Dabauvalle M-C van de Linde S . Rapidstorm: Accurate, fast open-source software for localization microscopy - Pubmed. Nat Methods. (2012) 9. doi: 10.1038/nmeth.2224 23132113

[24] Chiu C-L Clack N community tn . Napari: A Python multi-dimensional image viewer platform for the research community. Microsc Microanal. (2022) 28. doi: 10.1017/S1431927622006328 41292463

[25] Doose S . Locan: A Python library for analyzing single-molecule localization microscopy data - Pubmed. Bioinf (Oxford England). (2022) 38. doi: 10.1093/bioinformatics/btac160 35298593

[26] Ebert V Eiring P Helmerich D Seifert R Sauer M Doose S . Convex hull as diagnostic tool in single-molecule localization microscopy - Pubmed. Bioinf (Oxford England). (2022) 38. doi: 10.1093/bioinformatics/btac700 36315073

[27] Eiring P Steinhardt MJ Bauer N Vogt C Munawar U Han S . Single-molecule localization microscopy reveals the molecular organization of endogenous membrane receptors. Sci Adv. (2026) 12. doi: 10.1126/sciadv.aea2310 41637495 PMC12871438

[28] Schwingen NR Meretuk L Aigner M Kretschmann S Scholz JK Gsottberger F . Frontiers | Universal high-sensitivity Car T-cell monitoring by targeting linker sequences. Front Immunol. (2026) 17. doi: 10.3389/fimmu.2026.1787951 41958667 PMC13057459

[29] Lopci E de Jong D Dercle L . Frontiers | Editorial: Enhancing Car T-cell therapy with imaging. Front Immunol. (2025) 16. doi: 10.3389/fimmu.2025.1705002 41445757 PMC12723512

[30] Ho J Wang L Liu Y Ba M Yang J Zhang X . Promoter usage regulating the surface density of Car molecules may modulate the kinetics of Car-T cells *in vivo* - Pubmed. Mol Ther Methods Clin Dev. (2021) 21. doi: 10.1016/j.omtm.2021.03.007 33869653 PMC8027690

[31] Tristán-Manzano M Maldonado-Pérez N Justicia-Lirio P Muñoz P Cortijo-Gutiérrez M Pavlovic K . Physiological lentiviral vectors for the generation of improved Car-T cells. Mol Ther Oncol. (2022) 25. doi: 10.1016/j.omto.2022.05.003 35694446 PMC9163403

[32] Walker AJ Majzner RG Zhang L Wanhainen K Long AH Nguyen SM . Tumor antigen and receptor densities regulate efficacy of a chimeric antigen receptor targeting anaplastic lymphoma kinase. Mol Ther. (2017) 25. doi: 10.1016/j.ymthe.2017.06.008 28676342 PMC5589087

[33] Sonia G Avery DP Carolyn S Anna W Tong D Prachi RP . Enhancing Car T cell persistence through Icos and 4-1bb costimulation. JCI Insight. (2018) 3. doi: 10.1172/jci.insight.96976 29321369 PMC5821198

[34] Zheng Z Chinnasamy N Morgan RA . Protein L: A novel reagent for the detection of chimeric antigen receptor (Car) expression by flow cytometry - Pubmed. J Transl Med. (2012) 10. doi: 10.1186/1479-5876-10-29 22330761 PMC3299624

[35] Zaninelli S Meli C Borleri G Quaroni M Pavoni C Gaipa G . Optimization and validation of *in vivo* flow cytometry chimeric antigen receptor T cell detection method using Cd19his indirect staining - Pubmed. Cytometry Part A J Int Soc For Anal Cytol. (2024) 105. doi: 10.1002/cyto.a.24796 37707318

[36] Caballero A Escribà-Garcia L Pujol-Fernández P Escudero-López E Ujaldón-Miró C Montserrat-Torres R . High Car intensity of expression confers enhanced antitumor effect against lymphoma without functional exhaustion - Pubmed. Cancer Gene Ther. (2023) 30. doi: 10.1038/s41417-022-00518-6 36031661

[37] Eyquem J Mansilla-Soto J Giavridis T van der Stegen S Hamieh M Cunanan K . Targeting a Car to the Trac locus with Crispr/Cas9 enhances tumour rejection - Pubmed. Nature. (2017) 543. doi: 10.1038/nature21405 28225754 PMC5558614

