# Supplementary material for: A universally applicable toolbox for single-molecule quantification of chimeric antigen receptors using linker-resolved dSTORM microscopy
Source: Front Immunol. 2026 Jul 15;17:1897225. doi: 10.3389/fimmu.2026.1897225 (PMC13416521; doi:10.3389/fimmu.2026.1897225)
Supplement: Supplementary file 1 [file DataSheet1.docx]

Supplementary Material

# Supplementary Methods

## Generation of human CAR-T cells

For the generation of human CAR-T cells, blood samples were collected from healthy donors obtained from leukocyte reduction chambers provided by the Department for Transfusion Medicine of the University Hospital Würzburg after written informed consent. Peripheral blood mononuclear cells (PBMCs) were obtained through density gradient centrifugation using Ficoll-Hypaque. CD4^+^ and CD8^+^ T cells were isolated with immunomagnetic beads (Miltenyi Biotec) and activated using anti-CD3/CD28 Dynabeads (Life Technologies) at a bead:cell ratio of 1:1. Two days later T cells were transfected with transposase and transposon vector using the 4D‑Nucleofector (Lonza). CAR-T cells were isolated by sorting for EGFRt and expanded over 10 days by co-culturing with irradiated PBMCs and TM-LCL feeder cells.

## Flow cytometry and data analysis

Data was collected on a Cytek® Northern Lights™ (Cytek® Bioscience) and analyzed using FlowJo V10.8.1 (FlowJo LLC). A complete list of fluorochrome-conjugated antibodies used in this study is provided in Supplementary Table S1 in Section 1.3. For self-labeling, the Whitlow linker antibody was conjugated as described in the Methods Section 2.6. In detail, 2x10^5^ cells per well were plated in a 96-well plate and washed with PBS. Dead cells were excluded using GhostDye™Red 710 (Cytek® Bioscience) in PBS (1:10,000; 20 min at 4 °C). Cells were subsequently incubated with Fc receptor blocking reagent (hTruStain FcX™, BioLegend) at 1:100 for 15 min at 4 °C and stained with fluorochrome-conjugated antibodies for extracellular markers for 20 min at 4 °C. Following staining, cells were washed four times with FACS buffer and resuspended in FACS buffer for acquisition. For intracellular cytokine and transcription factor staining, cells were stimulated with phorbol myristate acetate (PMA)/Ionomycin (Sigma-Aldrich) for 4 h. T cells were subsequently washed and stained for cell surface markers, then fixed and permeabilized with the eBioscience™ Foxp3/Transcription factor staining buffer set (Thermo Fisher Scientific) according to the manufacturer’s protocol. For heatmap visualization of flow cytometry data, geometric median fluorescence intensity (gMFI) values for each parameter were extracted from FlowJo following standardized gating. To achieve comparative visualization across donors and constructs, marker expression values were Z-score normalized in GraphPad Prism 9 (GraphPad Software) per marker across all samples. Unsupervised hierarchical clustering and heatmap generation was performed using Euclidean distance and average linkage in Morpheus (Broad Institute, https://software.broadinstitute.org/morpheus).

## Linker antibody conjugation for CAR detection

Purified Whitlow antibody was purchased from Miltenyi Biotec (130-137-308). NaHCOH3 powder (Thermo Fisher Scientific; A17005.36) was prepared as a solution in dH2O with a concentration of 200 mM. Zeba Spin Desalting Columns (Thermo Fisher Scientific; A57759 MWCO:40K) were centrifuged three times at 2000 g for 1 min with 200 mM NaHCOH3 solution and supernatant was discarded. Purified antibody solution was transferred into buffer exchange columns and centrifuged at 2000 g for 2 min. Centrifuged antibody solution was mixed with Alexa Fluor 647 dye solution (Thermo Fisher Scientific; A20006, 1 mg/mL stock concentration in DMSO) and incubated at room temperature in the dark for 3 h. Afterwards, a new column was centrifuged three times at 2000 g for 1 min with PBS. The conjugated antibody solution was transferred into PBS washed columns and centrifuged at 2000 g for 2 min to remove unbound dye. The concentration and degree of labeling of the washed conjugated antibody solution was determined via UV-Vis spectroscopy using a Nanodrop^™^ (Thermo Fisher Scientific).

# Supplementary Figures and Tables

## Supplementary Figures

**
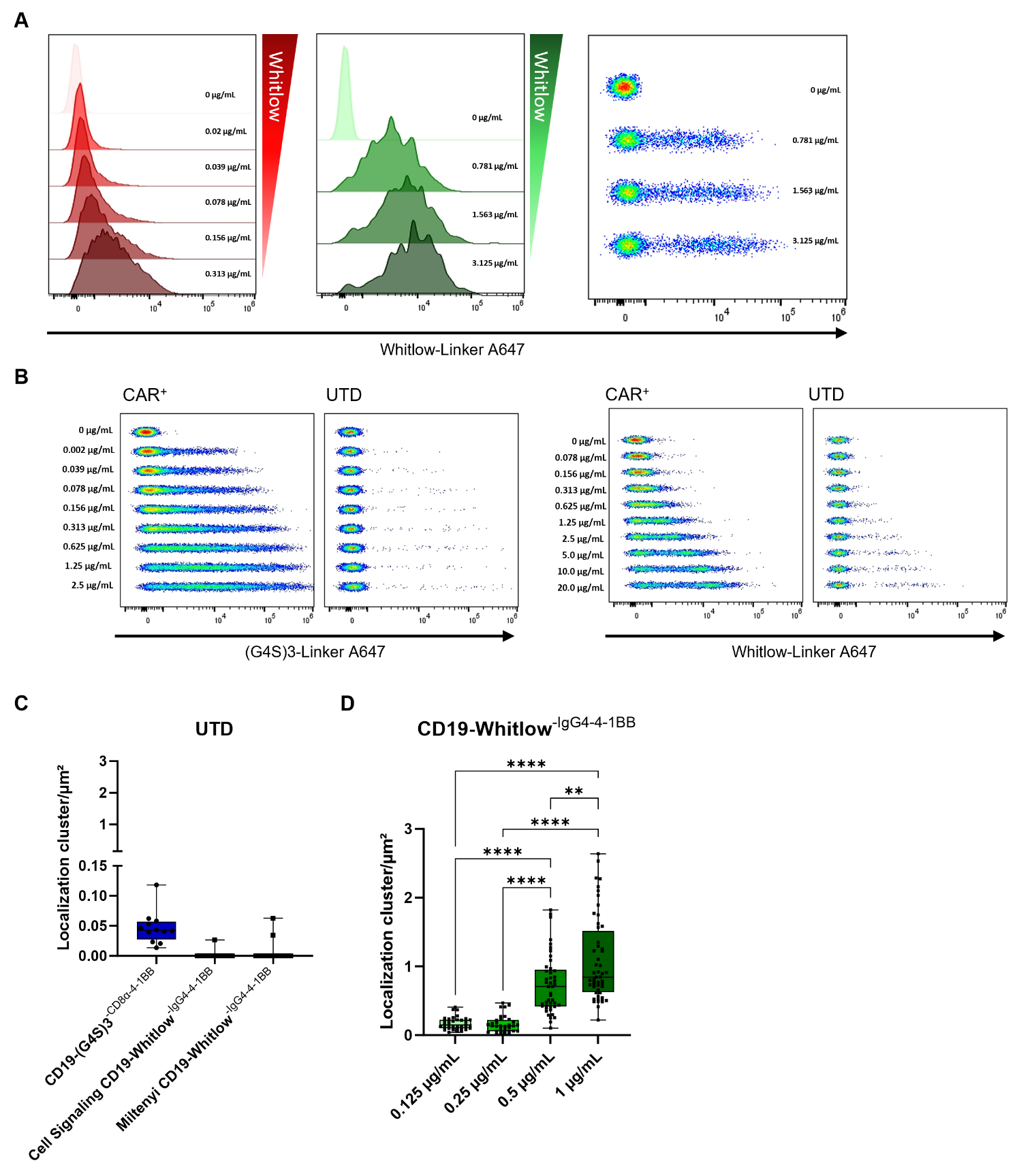
**

**Supplementary Figure 1:** Direct quantification of CAR surface expression by linker-specific staining using flow cytometry and *d*STORM. **A** Antibody titration for linker-specific detection of CAR construct by spectral flow cytometry. Representative histograms from one donor showing staining of EGFRt-sorted CAR-T cells or concatenated total cells stained with the commercially available Alexa Fluor 647-conjugated antibody targeting Whitlow linker (Cell Signaling; E3U7Q). **B**Density plots showing antibody titration on EGFRt-sorted CAR-T cells or untransfected T cells (UTD) stained using Alexa Fluor 647-conjugated antibodies targeting the (G4S)3- (left; Cell Signaling Technology, E7O2V) or Whitlow (right; Miltenyi Biotec, REA1400) linker. **C** *d*STORM-based quantification of CAR nanoscale organization of untransfected (UTD) CD8⁺ T cells stained with (G4S)3 (Cell Signaling Technology, E7O2V) or Whitlow linker (Miltenyi Biotec, REA1400) antibodies to assess nonspecific background signal. Each point represents an individual cell from one of three independent donors. Box plots indicate the median and interquartile range, with whiskers showing the minimum to maximum values. **D** Antibody titration (Cell Signaling, E3U7Q) for Whitlow-specific CAR detection by *d*STORM super-resolution microscopy. Each point represents an individual cell from one representative donor. Box plots indicate the median and interquartile range, with whiskers showing the minimum to maximum values. Statistical analysis was performed using Brown-Forsythe and Welch ANOVA with Welch correction. Significance indicated as: **** = P ≤ 0.0001; ** = P ≤ 0.01.


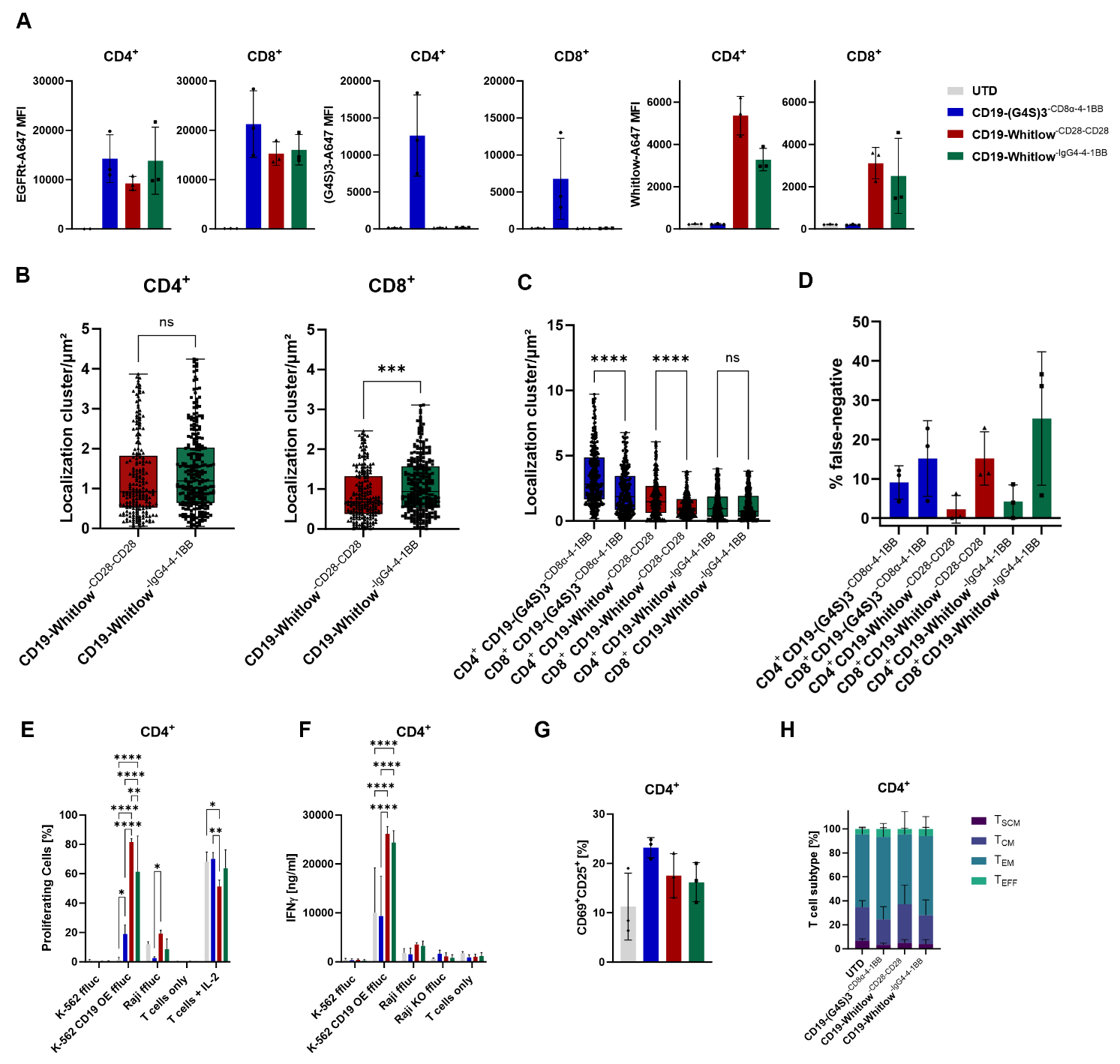


**Supplementary Figure 2:** Distinct CAR designs and receptor densities shape functional responses and phenotypic states in CD19 CAR-T cells. **A** EGFRt, (G4S)3, and Whitlow linker expression shown as MFI in CD4^+^ and CD8^+^ T- and CAR-T cells across the indicated groups. Data are presented as mean ± SD for n = 3 independent donors. **B** *d*STORM-based quantification of CD4^+^ and CD8^+^ CAR nanoscale organization using Whitlow-specific antibody staining (Cell Signaling, E3U7Q). Each point represents an individual cell from one of three independent donors. Box plots indicate the median and interquartile range, with whiskers showing the minimum to maximum values. Statistical analysis was performed using Brown-Forsythe and Welch ANOVA with Welch correction. **C** Comparison of nanoscale organization in CD4^+^ and CD8^+^ CAR-T cells determined by *d*STORM using the (G4S)3- (Cell Signaling Technology, E7O2V) or Whitlow (Miltenyi Biotec, REA1400) linker antibody. Each point represents an individual cell. Boxed-line indicates the median. Statistical analysis was performed using Brown-Forsythe and Welch ANOVA with Welch correction. **D** Percentage of false negative events obtained by subtracting the fraction of linker-negative cells identified by *d*STORM from the corresponding linker-negative fraction measured by flow cytometry. **E** Proliferation of CD4^+^ CD19 CAR-T cells compared with untransfected (UTD) T cells after 72 h co-culture with indicated tumor cells (E:T 4:1) shown as percentage of proliferating cells and representative histograms. **F** IFNγ secretion after 24 h co-culture of CD4^+^ CD19 CAR-T cells or untransfected (UTD) T cells with CD19^+^ tumor cell lines. **G** Activation shown as frequency of CD25^+^ CD69^+^ double-positive CD4^+^ CD19 CAR-T cells or untransfected (UTD) T cells. **H** Relative frequencies of CAR-T cell phenotypes for CD4^+^ CD19 CAR-T cells or untransfected (UTD) T cells. Phenotypes are classified as TSCM (stem cell memory-like), TCM (central memory-like), TEM (effector memory-like) and TEFF (effector-like). Significance indicated as: **** = P ≤ 0.0001; *** = P ≤ 0.001; ** = P ≤ 0.01; * = P ≤ 0.05; ns = P > 0.05.

## Supplementary Tables

Supplementary Table S1: Antibodies used for flow cytometry.

| Specificity | Fluorochrome | Clone | Dilution | Supplier | Catalog # |
| --- | --- | --- | --- | --- | --- |
| CD3 | BV785 | OKT3 | 1:800 | BioLegend | 317330 |
| CD4 | PerCP-Cy5.5 | OKT4 | 1:100 | BioLegend | 317428 |
| CD4 | BV750 | SK3 | 1:200 | BioLegend | 344644 |
| CD8 | BV605 | RPA-T8 | 1:100 | BioLegend | 301040 |
| CD279 | BV421 | EH12.2H7 | 1:25 | BioLegend | 329920 |
| TIGIT | BV480 | TgMab-2 | 1:50 | BD Biosciences | 570446 |
| TNFα | BV650 | W19063E | 1:1200 | BioLegend | 376212 |
| IFNγ | BV711 | 4S.B3 | 1:800 | BD Biosciences | 564793 |
| CD223 | BV785 | 11C3C65 | 1:200 | BioLegend | 369322 |
| Granzyme B | PB | GB11 | 1:800 | BioLegend | 515408 |
| CD69 | VioGreen | REA824 | 1:50 | Miltenyi Biotec | 130-112-611 |
| TOX | VioB515 | REA473 | 1:200 | Miltenyi Biotec | 130-129-208 |
| IL-2 | PE | MQ1-17H12 | 1:100 | BioLegend | 500307 |
| CD73 | PE-Dazzle594 | AD2 | 1:50 | BioLegend | 344020 |
| CD39 | Pe-Cy5 | A1bv | 1:800 | BioLegend | 328248 |
| CD25 | Pe-Cy7 | BC96 | 1:200 | BioLegend | 302612 |
| CD366 | APC-Cy7 | F38-2E2 | 1:100 | BioLegend | 345026 |
| CD45RA | BV711 | HI100 | 1:200 | BioLegend | 304138 |
| CD45RO | BV650 | UCHL1 | 1:20 | BioLegend | 304204 |
| CD62L | PE-Dazzle 594 | DREG56 | 1:80 | BioLegend | 304842 |
| EGFRt | A647 | 225 | 1:200 | Eli Lilly | NDC 66733-948-23 |
| (G4S)3 Linker | A647 | E7O2V | 1:20 | Cell Signaling Technology® | 69782S |
| Whitlow/218 Linker | A647 | E3U7Q | 1:2 | Cell Signaling Technology® | 69310S |
| Whitlow/218 Linker | A647 (self-conjugated) | REA1400 | 1:20 | Miltenyi Biotec | 130-137-308 |
